# Juvenile Myelomonocytic Leukemia in Turkey: A Retrospective Analysis of Sixty-five Patients

**DOI:** 10.4274/tjh.2017.0021

**Published:** 2018-03-06

**Authors:** Özlem Tüfekçi, Ülker Koçak, Zühre Kaya, İdil Yenicesu, Canan Albayrak, Davut Albayrak, Şebnem Yılmaz Bengoa, Türkan Patıroğlu, Musa Karakükçü, Ekrem Ünal, Elif Ünal İnce, Talia İleri, Mehmet Ertem, Tiraje Celkan, Gül Nihal Özdemir, Nazan Sarper, Dilek Kaçar, Neşe Yaralı, Namık Yaşar Özbek, Alphan Küpesiz, Tuba Karapınar, Canan Vergin, Ümran Çalışkan, Hüseyin Tokgöz, Melike Sezgin Evim, Birol Baytan, Adalet Meral Güneş, Deniz Yılmaz Karapınar, Serap Karaman, Vedat Uygun, Gülsun Karasu, Mehmet Akif Yeşilipek, Ahmet Koç, Erol Erduran, Berna Atabay, Haldun Öniz, Hale Ören

**Affiliations:** 1 Dokuz Eylül University Faculty of Medicine, Department of Pediatric Hematology, İzmir, Turkey; 2 Gazi University Faculty of Medicine, Department of Pediatric Hematology, Ankara, Turkey; 3 Ondokuz Mayıs University Faculty of Medicine, Department of Pediatric Hematology, Samsun, Turkey; 4 Erciyes University Faculty of Medicine, Department of Pediatric Hematology and Oncology, Kayseri, Turkey; 5 Ankara University Faculty of Medicine, Department of Pediatric Hematology and Oncology, Ankara, Turkey; 6 İstanbul University Cerrahpaşa Faculty of Medicine, Department of Pediatric Hematology and Oncology, İstanbul, Turkey; 7 Kocaeli University Faculty of Medicine, Department of Pediatric Hematology, Kocaeli, Turkey; 8 Ankara Children’s Hematology and Oncology Training and Research Hospital, Ankara, Turkey; 9 Akdeniz University Faculty of Medicine, Department of Pediatric Hematology and Oncology, Antalya, Turkey; 10 Dr. Behçet Uz Children Training and Research Hospital, Clinic of Pediatric Hematology and Oncology, İzmir, Turkey; 11 Necmettin Erbakan University Meram Faculty of Medicine, Department of Pediatric Hematology, Konya, Turkey; 12 Uludağ University Faculty of Medicine, Department of Pediatric Hematology, Bursa, Turkey; 13 Ege University Faculty of Medicine, Department of Pediatric Hematology, İzmir, Turkey; 14 Şişli Hamidiye Etfal Training and Research Hospital, Clinic of Pediatric Hematology and Oncology, İstanbul, Turkey; 15 Bahçeşehir University Faculty of Medicine, Department of Pediatric Hematology and Oncology, İstanbul, Turkey; 16 Marmara University Faculty of Medicine, Department of Pediatric Hematology and Oncology, İstanbul, Turkey; 17 Karadeniz Technical University Faculty of Medicine, Department of Pediatric Hematology and Oncology, Trabzon, Turkey; 18 Tepecik Training and Research Hospital, Clinic of Pediatric Hematology and Oncology, İzmir, Turkey

**Keywords:** Hematopoietic stem cell transplantation, Juvenile myelomonocytic leukemia, Turkey

## Abstract

**Objective:**

This study aimed to define the status of juvenile myelomonocytic leukemia (JMML) patients in Turkey in terms of time of diagnosis, clinical characteristics, mutational studies, clinical course, and treatment strategies.

**Materials and Methods:**

Data including clinical and laboratory characteristics and treatment strategies of JMML patients were collected retrospectively from pediatric hematology-oncology centers in Turkey.

**Results:**

Sixty-five children with JMML diagnosed between 2002 and 2016 in 18 institutions throughout Turkey were enrolled in the study. The median age at diagnosis was 17 months (min-max: 2-117 months). Splenomegaly was present in 92% of patients at the time of diagnosis. The median white blood cell, monocyte, and platelet counts were 32.9x10^9^/L, 5.4x10^9^/L, and 58.3x10^9^/L, respectively. Monosomy 7 was present in 18% of patients. JMML mutational analysis was performed in 32 of 65 patients (49%) and PTPN11 was the most common mutation. Hematopoietic stem cell transplantation (HSCT) could only be performed in 28 patients (44%), the majority being after the year 2012. The most frequent reason for not performing HSCT was the inability to find a suitable donor. The median time from diagnosis to HSCT was 9 months (min-max: 2-63 months). The 5-year cumulative survival rate was 33% and median estimated survival time was 30±17.4 months (95% CI: 0-64.1) for all patients. Survival time was significantly better in the HSCT group (log-rank p=0.019). Older age at diagnosis (>2 years), platelet count of less than 40x10^9^/L, and PTPN11 mutation were the factors significantly associated with shorter survival time.

**Conclusion:**

Although there has recently been improvement in terms of definitive diagnosis and HSCT in JMML patients, the overall results are not satisfactory and it is necessary to put more effort into this issue in Turkey.

## Abstract (tr)

**Amaç:**

Türkiye’deki juvenil miyelomonositik lösemi (JMML) hastalarının durumunu, tanı zamanı, klinik özellikler, mutasyon çalışmaları, klinik gidiş ve tedavi stratejileri açısından ortaya koymaktır.

**Gereç ve Yöntemler:**

Ülkemizdeki pediatrik hematoloji ve onkoloji kliniklerinden veri istenerek, JMML tanısı ile takip ve tedavisi yapılan hastaların klinik ve laboratuvar bulguları geriye dönük olarak değerlendirildi.

**Bulgular:**

On sekiz merkezden, 2002-2016 tarihleri arasında JMML tanısı alan toplam 65 hasta çalışmaya dahil edildi. Ortanca tanı yaşı 17 ay idi (2-117 ay). Splenomegali tanıda %92 hastada vardı. Ortanca lökosit, monosit ve trombosit sayıları sırasıyla 32,9x10^9^/L, 5,4x10^9^/L ve 58,3x10^9^/L idi. Monozomi 7, %18 hastada saptanmıştı. JMML mutasyonları 32 hastada (%49) çalışılmış olup, en sık rastlanan mutasyon PTPN11 idi. Hematopoetik kök hücre nakli (HKHN) hastaların ancak %44’üne uygulanabilmiş olup, nakillerin büyük bir oranı 2012 yılından sonra yapılmıştı. Nakil yapılamamasının en sık nedeni uygun donör bulunamamasıydı. Tanı aldıktan nakile kadar geçen ortalama süre 9 ay (2-63 ay) olarak saptandı. Tüm hastalarda 5 yıllık kümülatif sağkalım oranı %33, ortanca tahmini yaşam süresi ise 30±17,4 ay (%95 CI: 0-64,1) olarak bulundu. Sağkalım süresi HKHN yapılan hastalarda anlamlı olarak daha uzundu (log-rank p=0,019). Tanıda 2 yaşın üstünde olmak, trombosit sayısının 40x10^9^/L’nin altında saptanması ve PTPN11 mutasyon varlığı yaşam süresini anlamlı olarak kısaltan faktörler olarak bulundu.

**Sonuç:**

Ülkemizde her ne kadar son dönemlerde JMML hastalarında kesin tanı ve HKHN açısından iyileşme kaydedilmiş olsa da genel sonuç tatminkar değildir ve bu konu ile ilgili daha fazla çaba göstermeye gerek vardır.

## Introduction

Juvenile myelomonocytic leukemia (JMML) is a chronic malignant myeloproliferative disease of early childhood [1]. The World Health Organization classifies JMML in the group of myelodysplastic/myeloproliferative disorders owing to both myelodysplastic and proliferative features of the disease [2]. It is a rare disease comprising 2%-3% of all pediatric leukemias with a yearly incidence of 1.2 per million children [3,4]. Symptoms and signs of the disease result from infiltration of different organs including the spleen, liver, lungs, and gastrointestinal tract by leukemic cells [5,6]. Affected children generally present at a median age of 1.8 years with pallor, fever, infection, skin bleeding, cough, skin rash, marked splenomegaly, and sometimes diarrhea [5,7]. Leukocytosis with marked monocytosis, circulating myeloid/erythroid precursors, varying degrees of myelodysplasia and thrombocytopenia in peripheral blood, and an elevated hemoglobin F (HbF) corrected for age are common findings that are important for diagnosis. Bone marrow aspirate findings are not diagnostic per se but rather supportive with the presence of hypercellularity, predominance of granulocytic cells, and fewer than 20% blasts [5,6,7,8,9,10,11]. Monosomy 7 is the major cytogenetic anomaly found in 20%-25% of patients [5,7]. 

The majority of genetic mutations identified in JMML cause pathologic activation of the RAS-RAF-MAPK signaling pathway. These genes include *NF1, KRAS, NRAS,* and* PTPN1*. *NF1* and *CBL* are found in approximately 90% of patients [1,6,8]. The advances that have been achieved in the molecular characterization of JMML are important not only in diagnosis, but also in the management and prognosis of the disease, addressing a crucial phenotype-genotype relationship [1,2,8,12,13]. Some mutation types have been associated with mild clinical phenotypes and spontaneous remission rates, but the disease follows an aggressive course in the majority of cases if not treated [8,13,14,15,16,17]. Chemotherapy approaches have not been successful; the only curative treatment known so far is hematopoietic stem cell transplantation (HSCT) [7,8,9,11].  

The characteristics of the disease together with problems in finding a suitable donor for HSCT make the disease management difficult, especially in a developing country. In this context, we aimed to define the status of JMML patients in Turkey in terms of time of diagnosis, clinical characteristics, mutational analysis, clinical course, and treatment strategies. We think that identifying the problems in the management of this specific group of patients will help us achieve better care for them by taking the necessary precautions.

## Materials and Methods

Sixty-five children with JMML diagnosed between 2002 and 2016 in 18 institutions throughout Turkey were enrolled in the study. The diagnosis of JMML was based on previously published criteria [2,18,19,20]. Data including patient and disease characteristics and transplantation outcome were collected by standardized questionnaires for each patient.  

Due to the retrospective nature of the study, several patients had some missing data for some of the parameters.

### Clinical Assessment

Data including age, sex, presenting symptoms at first diagnosis, presence of recurrent fever, respiratory and gastrointestinal problems, rash, hepatosplenomegaly, and additional findings in physical examination were all noted. 

The details of the management of the disease for each patient including chemotherapy and HSCT were all noted.

### Laboratory Measurements, Bone Marrow, and Genetic Studies

Hematologic data including initial complete blood count, hemoglobin electrophoresis, analysis of peripheral blood smears, and bone marrow aspiration slides as well as cytogenetic and molecular genetic studies from the bone marrow aspirates were all noted. 

JMML mutations including *PTPN11, NRAS, KRAS*, and *CBL* were all studied at the University of Freiburg in the European Working Group on Myelodysplastic Syndromes in Childhood (EWOG-MDS) center. Analyses for *CBL* mutations were started after the year 2011. 

### Statistical Analysis

All statistical analyses were performed using SPSS 22 (IBM Corp., Armonk, NY, USA). Overall survival for all patients was defined as the time from diagnosis to death or last follow-up. Survival probabilities were estimated by Kaplan-Meier method and comparisons between different patient groups were performed using two-sided log-rank tests. Prognostic factors for the length of survival were analyzed by using the log-rank chi-square test. The choice of variables tested was based on our own results and other studies, and p<0.05 was considered statistically significant.

## Results

**Figure 1:**
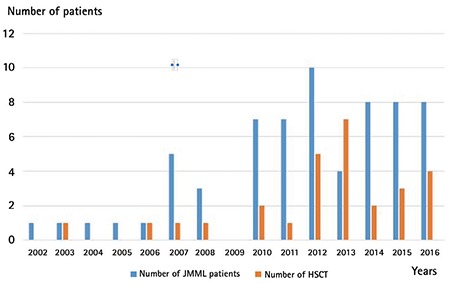
The distribution of newly diagnosed juvenile myelomonocytic leukemia patients and the number of juvenile myelomonocytic leukemia patients for whom hematopoietic stem cell transplantation was performed according to years. *JMML: Juvenile myelomonocytic leukemia, HSCT: hematopoietic stem cell transplantation.*

A total of 65 children were enrolled in the study. Only six had received the diagnosis of JMML between 2002 and 2006. The majority of the study patients (92%) had received the diagnosis of JMML in the last 10 years (2007-2016); 52 of them (80%) received the diagnosis after the year 2010 (Figure 1). 

### Clinical Features

The clinical characteristics of the patients are detailed in Table 1. 

The median age at diagnosis was 17 months (min-max: 2-117 months). Only three patients (4%) were older than 5 years old, the eldest being 9.7 years old. There was a male predominance with a male/female ratio of 2.25:1. The most common symptom at presentation was fever, followed by frequent infection, recurrent pulmonary symptoms, abdominal distension, and skin rash. Pallor was a presenting symptom in only 12% of patients.

Splenomegaly was present in 92% at the time of diagnosis, whereas lymphadenopathy was noted in 18%. Four children (6%) had the clinical diagnosis of neurofibromatosis type 1.  

### Laboratory Features

The hematologic data are given in Table 2. The median white blood cell (WBC), monocyte, and platelet counts were 32.9x10^9^/L, 5.4x10^9^/L, and 58.3x10^9^/L, respectively. The hemoglobin level was below 10 g/dL in 55 (84%) patients. 

The percentage of blasts in the bone marrow was less than 5% in the majority (69%) of patients. 

### Cytogenetics and JMML Mutation Analysis

Cytogenetic study of the bone marrow was available in 49 patients; of those, 9 patients (18%) were found to have monosomy 7 positivity (Table 2). Complex karyotypes were seen in three patients. The remaining 37 patients (75%) had normal karyotypes. 

JMML mutation analysis was performed in 32 of 65 patients (49%). The most common mutation was *PTPN11*, followed by *NRAS*,* KRAS*, and *CBL*, respectively (Table 3). Seven patients were found to have none of the screened mutations. The mutations were all somatic, except for one germline *CBL* mutation.

### Treatment Strategies

Treatment, either in the form of mild cytoreductive or acute myeloid leukemia (AML)-like intensive chemotherapy, was given to 46 of 61 patients (75%) with or without subsequent HSCT. The combination of low-dose cytarabine (40 mg/m^2^/day) and 6-mercaptopurine was the most frequent treatment given to 11 patients (23%), followed by high dose cytarabine+etoposide in 7 patients (16%) and 6-mercaptopurine in 6 patients (15%). The other less commonly used agents in treatment were azacitidine, cis-retinoic acid, hydroxyurea, and cytarabine, alone or in various combinations.  

HSCT was planned for 63 of 65 patients but could only be performed in 28 (44%) patients. Five other patients (8%) were also found to have suitable donors, but they were still waiting for HSCT at the time of data collection. The most frequent reason for not performing HSCT was the inability to find a suitable donor (21 patients: 33%). Patient/family incompatibility in 5 patients (8%) and death due to disease in 4 patients while planning HSCT (6%) were other reasons for not performing HSCT. 

“Watch and wait” was the main treatment strategy for those two patients for whom HSCT was not planned. One of them was a female patient with *CBL* mutation and the other was a male patient with *NRAS* mutation. 

The median time from diagnosis to HSCT was 9 months (min-max: 2-63 months).  

HSCT was performed in 28 patients (44%). Three patients were transplanted twice and two patients were transplanted three times due to relapse. The distribution of the transplanted patients according to years is shown in Figure 1. HSCT was performed for 50% of the newly diagnosed JMML patients after the year 2012. Donor type was matched sibling donor in 18 patients (64%), matched unrelated donor in 8 patients (28%), haploidentical donor in one patient, and unrelated cord blood in one patient.

### Survival

**Figure 2:**
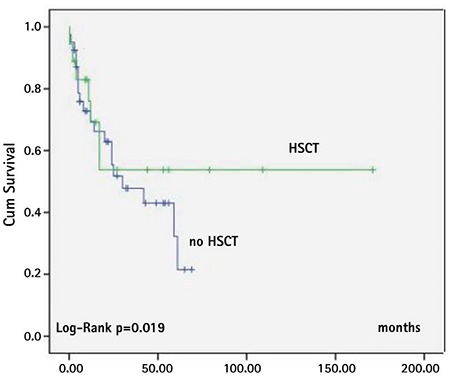
Survival of patients with or without hematopoietic stem cell transplantation (patients with hematopoietic stem cell transplantation: n=25, patients without hematopoietic stem cell transplantation: n=40). *HSCT: Hematopoietic stem cell transplantation.*

 The 5-year cumulative survival rate of the whole group was 33%. The mean estimated survival time was 72.4±12.9 months (95% CI: 46.9-97.9) and median estimated survival time was 30±17.4 months (95% CI: 0-64.1) for all patients. Survival time was significantly better in the HSCT group (log-rank p=0.019) (Figure 2). Relapse after HSCT occurred in 10 of 28 (35%) patients. Death occurred in 31 of 62 patients (50%); of those, 12 were in the HSCT (44%) group and 19 (54%) were in the non-HSCT group. The causes of death were HSCT toxicity (50%) and sepsis/organ failure due to relapse (50%) in the HSCT group. In all patients in the non-HSCT group, the cause of death was sepsis/organ failure due to progressive disease. 

### Factors Influencing Survival

**Figure 3:**
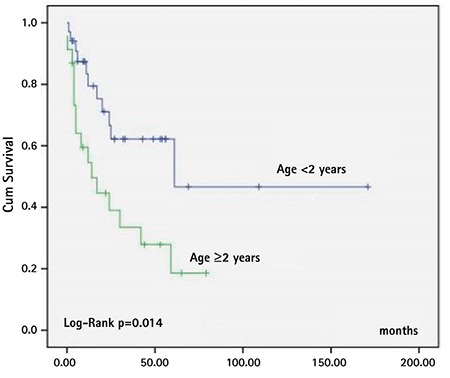
Survival of patients according to age at diagnosis (age <2 years: n=38 , age ≥2 years: n=27).

**Figure 4:**
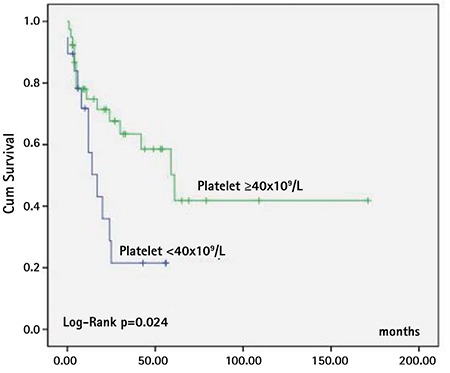
Survival of patients according to platelet count at diagnosis (platelets <40x10^9^/L: n=21, platelets ≥40x10^9^/L: n=44).

**Figure 5:**
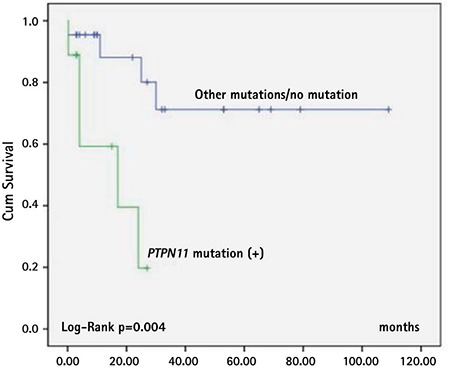
Survival of patients according to PTPN11 mutation status in patients for whom JMML mutation analysis was conducted (n=32) (PTPN11 mutation: n=9, NRAS/KRAS/CBL/no mutation: n=23).

Older age at diagnosis (>2 years old), platelet count at diagnosis of less than 40x10^9^/L, and *PTPN11* mutation were the factors associated with shorter survival time (Figures 3, 4, and 5; Table 4). Sex, fetal hemoglobin (HbF) percentage (<10% or ≥10%), presence of monosomy 7, and bone marrow blast percentage at diagnosis did not influence survival significantly (Table 4). 

## Discussion

This retrospective clinical study reflects the diagnosis, treatment strategies, and prognosis of 65 JMML patients from Turkey. The clinical features of the patients were highly similar to those reported in the literature [3,5,10,12]. In our study, the median time of diagnosis was found as 17 months and almost all of the patients (95%) were younger than 5 years old. The median age of diagnosis in the previous studies was reported within a range of 17-24 months old and more than 90% of patients were reported to be younger than 5 years old. The male predominance that was reported in other studies has also been observed in our study with a male:female ratio of 2.2 [4,5,7,12,21]. 

Patients with JMML have been commonly reported to present with symptoms of pallor, fever, infection, skin bleeding, cough, skin rash, and sometimes diarrhea [5,7]. The major presenting symptoms were fever and recurrent infection in the present study. Recurrent pulmonary infections and gastrointestinal symptoms were also seen in a substantial number of patients in this study. However, pallor was not a common symptom, which was reported as the major frequent symptom in the EWOG-MDS study [5]. In fact, the median hemoglobin level was 8.1 g/dL in our study and 84% of patients had an initial hemoglobin value of less than 10 g/dL. This was a retrospective study collecting data from patients’ records and so pallor might have been overlooked.

The presence of splenomegaly is a hallmark in the diagnosis of JMML; nevertheless, it has been reported that 7% of patients do not have splenomegaly at the time of diagnosis [6]. Similarly, splenomegaly was present in 92% of our patients at the time of diagnosis. Lymphadenopathy, on the other hand, was not as frequent in our patients as reported by the EWOG-MDS study [5]. Neurofibromatosis type 1 has been well recognized to have a 200- to 500-fold increased risk for development of JMML and has been reported in 8%-14% of JMML patients [5,10,22,23,24]. It was present in 4 patients (6%) in our study. 

Patients with JMML generally present with leukocytosis, monocytosis, and thrombocytopenia [5,6,7,8]. The hematologic data in our study were highly similar to those reported in the literature [5,7,12]. In our study, the median WBC, monocyte, and platelet counts were 32.9x10^9^/L, 5.4x10^9^/L, and 58.3x10^9^/L, respectively. The median HbF value was 8%. Locatelli et al. [7] reported the median WBC, monocyte, and platelet counts as 34x10^9^/L, 5.5x10^9^/L, and 65x10^9^/L, respectively, and the HbF value as 9%. These data show that although there might be some differences in the clinical presentation, hematologic data do not differ significantly among JMML patients. 

Major advances have been achieved in defining the genomic landscape of JMML in recent years [1,6,11,12,13,14,15,16,17]. Progress in the discovery of the underlying mutations helped in the definitive diagnosis of the patients and also led physicians to establish a phenotype-genotype relationship, predict the clinical outcome, and determine a treatment strategy. In JMML, for patients with *NF1* and somatic mutations of *PTPN11* and* K-RAS, *and for the majority of patients with somatic* NRAS *mutations, HSCT is recommended as the first treatment option [8]. As patients with germline *CBL* and a few patients with somatic *NRAS* mutations were reported to have had spontaneous remission, careful follow-up rather than HSCT is recommended in the first place for those patients [8,14,15,16,17]. In our study, mutational analysis was done for only 32 patients (49%) at the EWOG-MDS center. *PTPN11* mutation was the most frequently seen mutation (28%) and was also associated with significantly lower survival rates compared to other mutations. Somatic *PTPN11* mutations constitute ~35% of all JMML mutations and in some series have been reported to be associated with lower survival rates compared to other mutations [12,25,26]. Given the fact that the majority of the patients in this study had the diagnosis of JMML after 2010, mutational analysis was possible for most of them. In this respect, we hope that this study increases the awareness of JMML among physicians in terms of diagnosis as well as mutational analysis in order to outline a treatment strategy and to start a donor screening program immediately for HSCT if indicated. 

The only curative treatment approach in JMML to date has been HSCT [7,8,27,28,29]. HSCT was planned for all but two patients but could only be performed in 44% of the patients and it was associated with better survival time compared to those who were not transplanted. Mild cytoreductive or AML-like intensive chemotherapy was given to the majority of patients. Approximately one-third of the patients lacked a suitable donor for transplantation. The median time from the time of diagnosis to HSCT has been reported as between 6 and 10 months in various studies [7,27,28]. It was 9 months in our study, comparable to other studies, but 6% of the patients died while waiting for HSCT. It seems that besides finding a suitable donor we also had problems in performing HSCT. However, Turkey has made great progress in stem cell transplantation in the recent years. Besides the tremendous increase in the number of well-equipped stem cell transplantation centers in the last 5 years, difficulties in finding suitable donors have been mainly overcome. A national bone marrow bank, called Turkey Stem Cell Coordination Center, was established by the Turkish Ministry of Health in 2014 and has reached a substantial number of volunteer donors over time [30]. Along with these developments, most of our patients had stem cell transplantation in the recent years. Indeed, much effort has been needed, as HSCT remains the only curative treatment for this disease. 

Factors associated with poor prognosis other than mutational status have been reported as older age at diagnosis (>2 years), platelet count of <33-40x10^9^/L, and increased HbF level at diagnosis [4,5]. Consistent with the literature, besides *PTPN11* mutation, age older than 2 years and platelet count of less than 40x10^9^/L were associated with lower survival rates in our study. Patients with HbF level greater than 10%, as well as male sex and higher bone marrow blast percentage (5%-20%) at diagnosis, seemed to have worse outcomes, but the statistical differences were not significant. 

The natural course of JMML is aggressive and the great majority of patients die if the disease is left untreated [4,5,10]. The 5-year overall survival rate in JMML patients has been reported as 30%-40% in older studies [4,10]. However, with HSCT, the EWOG-MDS study reported the 5-year probability of overall survival as 64%, with the median observation time of patients alive being 40 months (min-max: 6-44) [7]. In our study, the 5-year cumulative survival rate of all patients was 33%, the median estimated time of survival was 30±17.4 months, and the most common cause of death was sepsis/organ failure due to progressive disease. This low survival rate in our patients obviously results from the low transplantation rate. Relapse after allogeneic stem cell transplantation has been a great problem in patients with JMML, occurring in one-third of transplanted patients [7,27,29]. The relapse rate was 35% in our study, and half of the relapsed patients had received more than one transplant. 

## Conclusion

In summary, the genotype-phenotype relationship becomes increasingly important in JMML. As a result, mutational analysis is important not only for definitive diagnosis of the disease but also to determine the indication and urgency for HSCT, and to promptly initiate donor screening if necessary. Although there is a possibility of spontaneous remission with certain types of mutations, HSCT still remains the only curative treatment for this disease. As the main reason for not performing HSCT was the inability to find a suitable donor in this study, we think that it is necessary to put more effort into this issue in Turkey.

## Figures and Tables

**Table 1:**
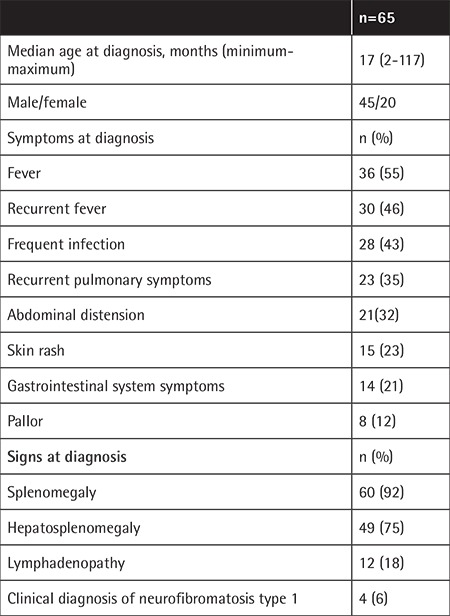
Clinical characteristics of the patients.

**Table 2:**
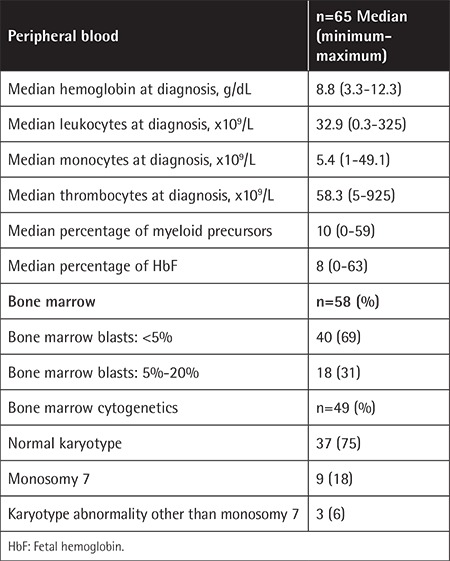
Hematologic data of the patients at diagnosis.

**Table 3:**
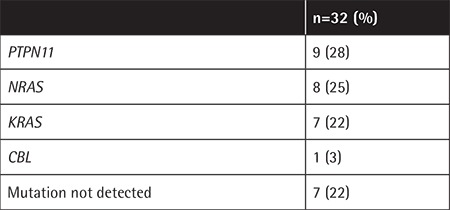
The distribution of juvenile myelomonocytic leukemia mutations in 32 patients.

**Table 4:**
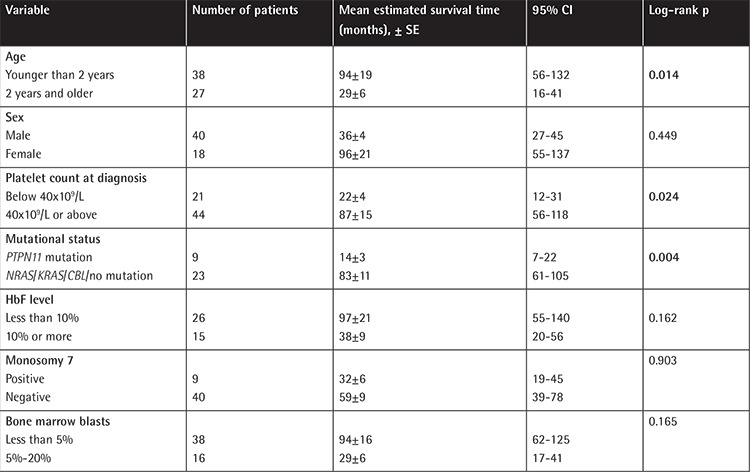
Factors influencing survival in patients with juvenile myelomonocytic leukemia.
